# Seq’ing identity and function in a repeat-derived noncoding RNA world

**DOI:** 10.1007/s10577-020-09628-z

**Published:** 2020-03-07

**Authors:** Rachel J. O’Neill

**Affiliations:** 1 Institute for Systems Genomics, University of Connecticut, Storrs, CT 06269 USA; 2 Department of Molecular and Cell Biology, University of Connecticut, Storrs, CT 06269 USA; 3 Department of Genetics and Genome Sciences, University of Connecticut Health Center, Farmington, CT 06030 USA

**Keywords:** noncoding RNA, chromatin-associating RNAs, R-loop, triplex, repeat annotation, transposable element (TE)

## Abstract

Innovations in high-throughout sequencing approaches are being marshaled to both reveal the composition of the abundant and heterogeneous noncoding RNAs that populate cell nuclei and lend insight to the mechanisms by which noncoding RNAs influence chromosome biology and gene expression. This review focuses on some of the recent technological developments that have enabled the isolation of nascent transcripts and chromatin-associated and DNA-interacting RNAs. Coupled with emerging genome assembly and analytical approaches, the field is poised to achieve a comprehensive catalog of nuclear noncoding RNAs, including those derived from repetitive regions within eukaryotic genomes. Herein, particular attention is paid to the challenges and advances in the sequence analyses of repeat and transposable element–derived noncoding RNAs and in ascribing specific function(s) to such RNAs.

## Introduction

Since the discovery of *Xist* (Brown et al. 1992), a long noncoding RNA that directs inactivation of the mammalian X chromosome, our understanding of the role RNAs play in chromosome biology has expanded well beyond the fundamental “RNA codes for proteins” dogma. The vast majority of RNAs produced by RNA polymerase II are mRNAs, and as such are capped and polyadenylated for subsequent transport outside of the nucleus, yet a surprising amount of RNA remains in the nucleus, where the bulk of RNA turnover occurs. These nuclear residents are incredibly diverse and include trimmed and spliced portions of pre-mRNAs, RNA debris from RNA decay, repeat-derived RNAs, antisense RNAs, and other forms of noncoding RNAs (ncRNA) (reviewed in (Nozawa and Gilbert 2019; Palazzo and Lee 2015)). In addition to simply being isolated from the translation pipeline, nuclear ncRNAs are in an environment where they can interact directly with DNA and/or chromatin and thus exert an influence over fundamental processes such as transcription and genome stability (Mattick 2001; Mattick 2005; Mattick 2009).

Early experiments indicated that ~ 10% of the mass of chromatin was RNA (Holmes et al. 1972), considered at that time to be part of the ribonucleoproteinaceous structures comprising a static “nuclear matrix” (Fey et al. 1986a; Fey et al. 1986b) supporting nuclear organization. Today, the idea of a static matrix has been abandoned (Pederson 2000) in favor of models invoking a dynamic nuclear organization of which RNA is an integral part. Since nuclear ncRNA content can vary across different cellular contexts, ncRNAs may serve as an architectural feature required for establishing specific chromatin states (Caudron-Herger and Rippe 2012; Mele and Rinn 2016), and thus foster a permutable form of control over genome organization (Michieletto and Gilbert 2019; Nozawa and Gilbert 2019). Additionally, sequence variation inherent to many ncRNAs, particularly repeat-derived ncRNAs, could provide a potent source of species-specific genome organization and evolutionary novelty (Hall and Lawrence 2016; Kapusta et al. 2013; Necsulea et al. 2014).

The capacity of RNAs to associate with chromatin, either through DNA or protein interactions, indicates they may act as molecular signals, regulators, guides and/or scaffolds (Chu et al. 2011; Guttman and Rinn 2012; Rinn et al. 2007). Moreover, they may contribute to the regulation of entire chromosomes, as *Xist* does*,* or specific chromosomal domains within a cell and thus may mediate specific cellular processes such as centromere function and chromosome inheritance (e.g., Carone et al. 2009; Carone et al. 2013; Topp et al. 2004; Wong et al. 2007) and thus foster chromosome evolution (Brown et al. 2012; Brown and O’Neill 2010; O’Neill and Carone 2009). Revealing the composition of RNAs that influence chromosome biology, defining how they interact with the genome and/or chromatin, and ascribing a cellular function, if any, to these interactions are among the grand challenges at the frontier of chromosome research.

These challenges are being met by innovations to high-throughput sequencing (HTS) approaches (a.k.a. the growing menagerie of “…-seq”s) to the study of RNA. Coupled with revolutionary advances in long-read sequencing, genome assembly, and annotation methods, comprehensive cataloging of nuclear ncRNA is underway with a view towards understanding the cellular functions of these heterogeneous and fascinating biomolecules. This review focuses on some of the recent technological developments that have enabled isolation of both chromatin-associated RNAs and DNA-associated RNAs. Moreover, computational approaches and initiatives to achieve chromosome-level genome assemblies are discussed in light of the challenges in studying such RNAs.

## How do we define ncRNA?

Given that we have known about ncRNAs in the nucleus for over 50 years, why has it been so challenging to ascribe reasons for their existence? The first challenge, and arguably one that has yet to be fully overcome, is clarity on how one defines the component of nuclear RNAs that are *noncoding;* in other words, what exactly is a ncRNA? The phrase “noncoding RNA” at face value could refer to any RNA molecule that does not lead to a translated protein. However, this would include spliced introns, degradation products, and RNA debris, as well as RNAs that are predictably transcribed and have a structured transcription unit, such as rRNAs and tRNAs. Current nomenclature distinguishes ncRNAs rather arbitrarily as either small RNAs of 200 nt and less, or RNAs 200 nt and longer, referred to as long or large ncRNAs (lncRNAs) and long intergenic noncoding RNAs (lincRNA). Small RNAs are further classified into groups based on function, biogenesis, and/or other biochemical features (e.g., snoRNAs, tRNAs, miRNAs, piRNAs, etc.) (Dupuis-Sandoval et al. 2015; Kim et al. 2009; Oberbauer and Schaefer 2018; Ozata et al. 2019; Pan 2018; Treiber et al. 2019).

Beyond the size designation of the larger ncRNAs fraction as > 200 nt, relatively little else classifies or distinguishes lncRNAs and for many, the full transcription unit has not been adequately annotated in genome assemblies. Of the few lncRNAs that have been heavily studied, the underlying transcription units are often quite long, such as the 2.3-kb *H19*, the first lncRNA annotated in human (Brannan et al. 1990), the ~ 8-kb MALAT1 (Tripathi et al. 2010), the 17-kb *Xist* (Brown et al. 1992), and the 2.2-kb HOTAIR (Rinn et al. 2007). These RNAs, along with a few other well characterized transcripts, are known to participate in specific cellular functions, such as splicing, translation, RNA editing, and transcription (see Qian et al. (2019) for a review). The overall length of these lncRNAs has no doubt facilitated their annotation in assembled and well-curated genomes (i.e., mouse and human), while smaller or more divergent lncRNAs have been more challenging to catalog and study.

## The road to defining ncRNA function

Recent comparative studies utilizing transcriptomic datasets and available genome assemblies have revealed a collection of lncRNAs with enough sequence conservation across species to enable at least partial annotations and functional inferences (Cabili et al. 2011; Guttman et al. 2009; Marques and Ponting 2009; Necsulea et al. 2014). However, the low sequence conservation among the vast majority of lncRNAs limits the ability to use sequence alone for annotation or to surmise functions. Further complicating the classification of lncRNAs is the observation that transposable element sequences (TEs) contribute to a significant portion of the lncRNA repertoire (Kapusta et al. 2013). In fact, TEs are ubiquitous in lncRNAs in vertebrates and account for a large fraction of total ncRNA sequences (Kapusta et al. 2013).

It is possible that the insertion of exonic portions of TEs into lncRNAs, termed repeat insertion domains of lncRNAs (RIDLs) (Johnson and Guigo 2014), represent exaptations of TE sequences (Johnson 2019). For example, a short sequence motif found in several unrelated lncRNAs was identified in human cells that increase nuclear enrichment through binding to HNRNPK (Lubelsky and Ulitsky 2018). This motif, SIRLOIN (SINE-derived nuclear RNA localization), overlaps with antisense sequences of the Alu SINE repeat element, indicating the nuclear-retention of RNAs mediated by this motif may be part of a pathway to regulate transcripts that contain Alu insertions (Lubelsky and Ulitsky 2018). Some TE insertions, however, may have limited or no impact on the function of a lncRNA and thus are simply not selected against, as is the case with lineage-specific TE insertions found in the *Xist* lncRNA (Kapusta et al. 2013). Alternatively, the first portion of many lncRNAs, and often the entire lncRNA itself, is comprised of TE sequences, indicating that TE insertions in genomic sequences can provide the transcription start site, and subsequently produce a new lncRNA (Kapusta et al. 2013). Thus, the divergence of genomic TE content across different lineages provides fodder for the recruitment of lineage-specific lncRNAs (Kapusta et al. 2013).

Further confounding the study of ncRNAs utilizing cross-species sequence comparisons is the fact that divergent, non-TE repeats are often expressed. Satellite repeats, for example, are a class of ncRNAs that is found in most eukaryotes (reviewed in (Biscotti et al. 2015; Hartley and O’Neill 2019; Talbert and Henikoff 2018)). Satellite-derived ncRNAs are produced from genomic loci that vary in composition from simple repeats consisting of a small number nucleotides organized in tandem arrays to longer satellite arrays of repeated units that are each 10’s to 1000’s of bases in length. In many cases, these ncRNA producing repeats are found in clusters in specific chromosome regions, such as large heterochromatin blocks on chromosome arms, centromeres, and telomeres, linking transcription of highly repetitive ncRNAs to chromosome function.

Given their abundance and diversity, teasing apart functional from non-functional ncRNAs has been challenging and even controversial (e.g., Graur et al. 2013; Palazzo and Lee 2015; Pennisi 2012). A series of commentaries highlight some of the problems with the use of the term “functional” when applied as a blanket descriptor to a ncRNA (Doolittle 2018; Laubichler et al. 2015; Palazzo and Lee 2015). The issues lie in the fact that “function” is interpreted differently in molecular (*what does the ncRNA do*) vs evolutionary (*why does the ncRNA exist*) contexts. Recently, a new lexicon to clarify “function” has been proposed, referred to as the “Pittsburg model of function”. In this model, ncRNAs are further classified into five categories based on the depth and context of genetic information available to support functional classification (Table 1) (Keeling et al. 2019). Such a refined framework for presenting data on ncRNAs is long overdue; the application of these categories offer clarity for the field as we navigate discoveries of the myriad chromatin-associated ncRNAs across different cell types and developmental stages, and particularly across different species (Doolittle 2018).

**Table 1:** The Pittsburgh Model of Function as it relates to describing the function of any given ncRNA. The functional classification beings with the defined occurrence of a ncRNA (*expression)* and sequentially increases in the level of the classification based on the type of functional information garnered from studying the ncRNA in its biological system

Classification/meaning	Definition
Vague	Insufficient evidence to infer one or more meanings of function within this model, nor to derive a new meaning
Expression	The presence or amount of ncRNA transcript
Capacities	Intrinsic physical properties of ncRNA; the necessity of the object’s behavior given an environment (e.g., structural constraints)
Interactions	Physical contacts, direct or indirect, between the ncRNA and the other components of a system
Physiological Implications	The ncRNA’s involvement in biological processes as enabled by a set of its capacities, interactions, and expression patterns, independent of cross-generational considerations.
Evolutionary Implications	The ncRNA’s influence on population dynamics over successive generations, as enabled by its physiological implications and their interplay with environmental pressures.

## Entering a new era of transcriptome profiling

Early genomics approaches that were designed to assess transcriptional output across different samples often employed exon-based screens (e.g., microarrays), ignoring repeat-derived and intergenic ncRNAs, thus rendering only a partial view of transcriptome dynamics. HTS approaches support transcriptome-scale sequencing (RNA-seq) that include ncRNAs by capturing potentially all RNAs present in a given sample, representing newly transcribed RNAs, stable RNAs, and RNAs heading for imminent decay. While RNA-seq was the first global transcriptomic approach enabled by HTS, new techniques have been developed to score the density of RNA polymerase II binding across the genome or to measure nascent, active transcription, and delineate transcription start sites (TSSs), eliminating the need to account for the variable half-life of different RNAs. Sequencing data outputs are subsequently mapped to a reference genome and intersected with gene annotations to tease apart mRNAs from cell-specific ncRNAs.

**Fig. 1:**
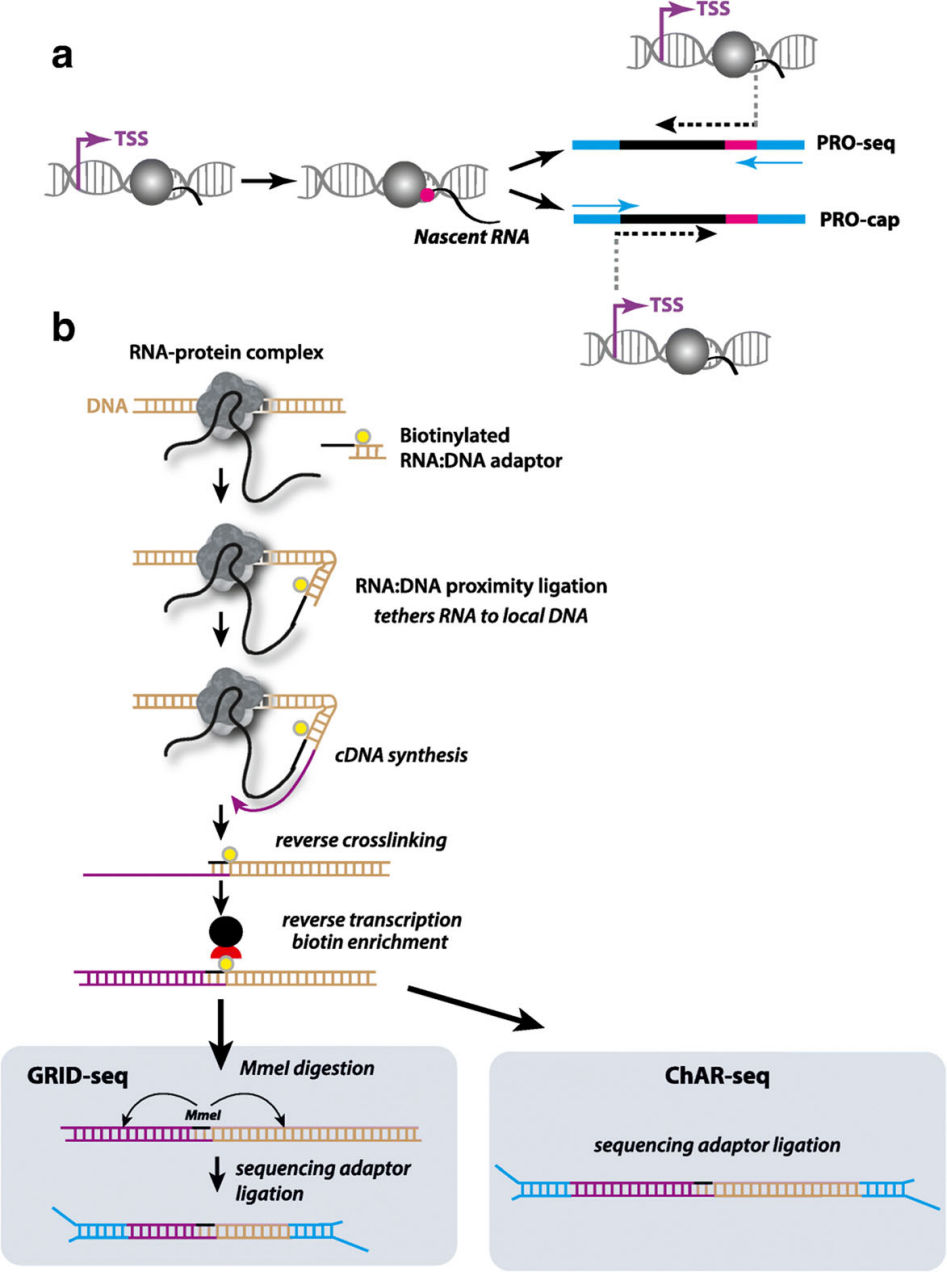
**a** Using a genome-wide nuclear run-on reaction incorporating a biotin-labeled ribonucleotide (pink) followed by sequencing adaptor (blue) ligation, PRO-seq (top) is used to capture sites of active RNA polymerase engagement and PRO-cap is used to identify transcription start sites (TSS). **b** Both GRID-seq and ChAR-seq start by cross linking RNA-protein-DNA complexes and proximity ligation of an RNA-DNA hybrid adaptor that is biotinylated (yellow). cDNA synthesis (purple) proceeds from the adaptor, resulting in sequences containing cDNA (purple), the biotinylated adaptor (black and yellow), and presumed interacting DNA sequence (tan). After reversal of crosslinks, proximity-ligation products are enriched using streptavidin-coated magnetic beads. GRID-seq (left) proceeds with *MmeI* digestion based on the *MmeI* recognition sequence within the adaptor. Following cleavage, which occurs ~ 20 bp from the hybrid adaptor, sequencing adaptors are ligated (blue) for subsequent HTS. ChAR-seq (right) does not rely on *MmeI* digestion, allowing for the capture of more sequence information following sequencing adaptor ligation (blue) and HTS

Immunoprecipitation of RNA polymerase II (Churchman and Weissman 2011; Larson et al. 2014; Nojima et al. 2015) and isolation of insoluble chromatin (Weber et al. 2014) have been used to identify nascent transcripts, revealing the involvement of nucleosome positioning in transcription elongation (Churchman and Weissman 2011). However, variation in antibody specificity or the efficiency of chromatin purification may affect experimental outcomes of such approaches (Mahat et al. 2016). Adaptations to nuclear run-on experiments (see (Smale 2009)) that enable genome-wide capture of nascent transcripts bypass immunoprecipitation, instead using labels incorporated into nascent RNA to isolate purified transcripts. In GRO-seq (global run-on sequencing), bromouridine is used to label nascent RNAs (Core et al. 2008); the incorporation of multiple labeled nucleotides in the run-on reaction allows a mapping resolution of 10’s of bases. In a modification of this technique, PRO-seq (precision run-on sequencing), biotin-labeled NTPs are added to the run-on reaction and nascent transcripts with an incorporated biotin-NTP are sequenced from the 3′ end to afford single-bp resolution of the site of RNA polymerase engagement with nascent RNA when mapped back to a reference genome (Fig. 1a) (Kwak et al. 2013; Mahat et al. 2016). PRO-cap, an adaptation of PRO-seq, incorporates steps to repair the 5′ end of the nascent transcript (i.e., capping) for adaptor ligation and subsequent sequencing from the 5′ end, providing TSS identification (Fig. 1a) (Kwak et al. 2013; Mahat et al. 2016). Further building upon the principle of PRO-seq is the recent development of ChRO-seq (chromatin run-on sequencing) (Chu et al. 2018), wherein the input material is not nuclei isolated from cells, but rather is fractionated, insoluble chromatin that includes engaged RNA polymerase II (Wuarin and Schibler 1994), increasing the diversity of samples that can be queried.

By uncovering nascent transcripts independent of innate transcript stability, a model of transcription initiation and elongation is emerging, revealing some of the fundamental signatures of RNA polymerase II activity. For example, promoters and enhancers share the genomic signal of divergent transcription profiles for nascent transcripts, but can be distinguished based on the transcription level and stability of the resulting transcripts (Core et al. 2008). From these observations, it appears that histone modifications that vary between promoters and enhancers are not necessarily dictated by the type of regulatory element at which they reside, but rather are associated with specific transcriptional signals. Revealing patterns of nascent transcription at the genome-scale is supporting more accurate annotations of regulatory regions and active transcription across different cell types/stages, independent of factors that can influence transcript abundance in the nucleus. Furthermore, ongoing efforts to capture a view of the changing transcriptional landscape among different tissues, conditions, developmental stages, and across different species is starting to reveal the true, and indeed extremely diverse and dynamic repertoire of ncRNAs. Understanding the fate of these ncRNAs and delineating whether the ncRNA sequence itself, the act of its transcription, or both, impact genome dynamics requires a combination of innovative tools to capture ncRNAs, delineate their interacting partners, and decipher their mode of function at the genome-scale.

## Looking beyond transcription for ncRNA partners

To begin to understand the ways in which ncRNAs may impact genomes at both local (gene transcription, local chromatin states) and regional (chromosomal regions and entire chromosomes) scales, one must consider *how* and *where* ncRNAs associate with chromatin beyond their site of nascent transcription (Guttman and Rinn 2012). ncRNAs can associate with chromatin in *cis* and/or *trans* through either direct RNA-DNA interactions or through an intermediary, such as chromatin-associated protein or protein complex. Different methods have been developed to tease apart ncRNAs based on these varied interactions. From these studies, we have not only begun to unravel the ncRNA-chromatin interactome, but have gained an appreciation for the varied, and in some cases, seemingly contradictory roles ncRNAs play in processes such as gene regulation, chromosome function, and genome organization.

### RNA:DNA partnerships—R-loop detection

Direct RNA-DNA interactions occur through complementary base pairing of DNA with RNA, resulting in the formation of a three-stranded structure consisting of a DNA:RNA hybrid and the displaced complementary DNA strand (Drolet et al. 1995; Thomas et al. 1976). Tiny, three-stranded “bubbles”occur during RNA-priming of DNA replication and at the immediate site of RNA polymerase as transcription occurs; longer, stable forms of these three-stranded structures are called R-loops (RNA moiety loop) (Thomas et al. 1976). R-loops were originally considered an extension of the RNA:DNA hybrid found within the RNA polymerase II transcription bubble (Westover et al. 2004), but it appears more likely that they result from the fold-back of nascent RNA as it exits the polymerase, known as an RNA thread back model (Roy et al. 2008).

In normal cells, an equilibrium is maintained that balances the formation and resolution of R-loops to support genome integrity (e.g., Chakraborty and Grosse 2011; El Hage et al. 2010; Zhou et al. 2014). Although R-loop formation has been linked to genome instability and disease (reviewed in (Santos-Pereira and Aguilera 2015)), R-loop structures may also serve important roles in normal cells. For example, R-loops facilitate the programmed immunoglobulin class switch recombination in B cells (Roy et al. 2008; Yu et al. 2003). Bolstered by computational predictions that R-loops could be prevalent across the genome (Ginno et al. 2012), genome-scale methods have been developed to identify R-loops and potentially reveal novel regulatory functions.

A genome-wide assessment of R-loops that form under normal cellular conditions was afforded by the development of an antibody (S9.6) to RNA:DNA duplex structures specifically, independent of nucleic acid sequence (Boguslawski et al. 1986). Immunopreciptation with the S9.6 antibody coupled with deep sequencing, a technique known as DRIP-seq (DNA:RNA immunoprecipitation coupled to high-throughput sequencing), results in a genome-wide map in R-loop sites in specific tissues (Ginno et al. 2012). Variations of this technique, including S1-nuclease DRIP-seq (S1DRIP) (Wahba et al. 2016), bisulfide DNA:RNA immunopreciptation (bis-DRIP) (Dumelie and Jaffrey 2017), and RNA:DNA immunoprecipitation (RDIP) (Nadel et al. 2015) have built upon the original DRIP-seq method to collectively develop preliminary maps for R-loop formation in specific cells. However, these techniques have some limitations in that the harsh preparation of the chromatin for immunoprecipitation may disrupt all but the most stable R-loops (Yan et al. 2019) and the S9.6 antibody may also recognize dsRNA (Hartono et al. 2018), complicating data interpretation.

Alternative methods employ a form of RNAse H, which has an affinity towards RNA:DNA heteroduplexes that is catalytically incapable of cleaving RNA. These methods, DRIVE (DNA:RNA in vitro enrichment) (Ginno et al. 2012) and R-ChIP (R-loop chromatin enrichment) (Chen et al. 2017), no longer rely on S9.6, overcoming doubts about the specificity of the antibody, but still suffer from challenges presented by the affinity purification steps. A method that no longer relies of affinity purification has been developed that is based on the cleavage under targets and release using nuclease (CUT&RUN) approach (Skene and Henikoff 2017) combined with RNAse H specificity for RNA:DNA heteroduplexes. This approach, MapR, revealed previously undetected transient R-loops at promoters and active enhancers (Yan et al. 2019).

Collectively, these types of approaches have revealed that R-loops are found in the terminators and enhancers of some genes, and thus can influence transcriptional control. For example, R-loops that form immediately following a transcription start site in a CpG island prevent DNA methylation of the underlying gene via DNA methyltransferase 3B1, thus facilitating transcription activation (Ginno et al. 2012). Moreover, the overlap between R-loops and GC-skew in the 5′ end of genes is also correlated with the deposition of histone marks of active transcription, including H3K4me3, H4K20me1, and H3K79me2 (Ginno et al. 2013; Ginno et al. 2012), implicating these R-loops as intermediaries in chromatin dynamics. R-loops may also function in transcript termination processes, such as RNA polymerase II pausing (Skourti-Stathaki et al. 2011) and induction of antisense transcription. When antisense transcripts are formed, these ncRNAs trigger dsRNA formation and the deposition of H3K9me2 and HP1γ, marks of repressive heterochromatin (Skourti-Stathaki et al. 2014). The ability of R-loops to trigger the formation of heterochromatin, histone H3 S10 phosphorylation, and chromatin condensation (Castellano-Pozo et al. 2013) may facilitate transcript silencing through establishment of repressive chromatin, but may also lead to replication fork stalls and DNA fragility/breakage (Castellano-Pozo et al. 2013; El Achkar et al. 2005; Groh et al. 2014).

R-loops, while largely considered in the context of *cis* ncRNA interactions, can be formed by *trans-*acting RNAs (Wahba et al. 2013), indicating that a single RNA species may affect many loci across the genome that share a similar sequence composition, such as repeated elements and satellite arrays. The single stranded DNA binding protein RPA (replication protein A) was recently identified at human centromeres. While RPA is known to participate in ATR (ataxia telangiectasia mutated and Rad3-related) kinase activation targeting DNA damage and stalled replication forks (Zou and Elledge 2003), normal centromeres do not appear to recruit RPA through damage response mechanisms (Minocherhomji et al. 2015). Instead, RPA is recruited by the single stranded DNA that is displaced in R-loops, indicating R-loops may be a general feature of centromeres (Kabeche et al. 2018). Indeed, staining with the S9.6 antibody indicates that R-loops are prevalent at human centromeres and their association with ATR activation implicates that the formation of R-loops may be required to for activation of Aurora B and accurate chromosome segregation (Kabeche et al. 2018). It is possible that nascent transcripts forming centromeric R-loops are acting *in cis*, facilitated by the repeat-derived transcripts produced in active centromeres (Carone et al. 2009; May et al. 2005; McNulty et al. 2017; Rosic et al. 2014; Ugarkovic 2005). Alternatively, centromeric R-loops may be mediated by a *trans*-activating ncRNA, perhaps recognizing the repeat motif present in CENP-B DNA binding sites shared across divergent and chromosome-specific centromeric satellites (Masumoto et al. 1989). As the genomic landscape of highly repeated regions such as centromeres become more accessible (see below), RNA-DNA and RNA-Chromatin sequencing approaches combined with innovative computational approaches offer promise in revealing the complex RNA interactions that mediate centromere function and chromosome stability.

### RNA:DNA partnerships—triplex detection

Without disrupting the hydrogen bonds of the DNA helix, RNA is still capable of direct nucleic acid interaction via the formation of a DNA:RNA triple helix (an RNA:DNA triplex, or simply “triplex” (Felsenfeld and Rich 1957) (not to be confused with the three strandedness of R-loops). A triplex forms when RNA binds to the major groove of a purine-rich stretch of duplex DNA through Hoogsteen or reverse Hoogsteen hydrogen bonding (reviewed in (Bacolla et al. 2015; Li et al. 2016)). Triplex formation has been shown to affect chromatin state through the recruitment of epigenetic modifiers, particularly when the interacting RNA in the triplex structure is a lncRNA. For example, local tethering of PRC to *Foxf1*, and subsequent trimethylation of histone 3 lysine 27 residues (H3K27me3), is mediated by a triplex containing the *Fendrr* lncRNA (Grote and Herrmann 2013). The ability of lncRNA-triplex structures to act as scaffold structures to recruit chromatin remodeling complexes (Bacolla et al. 2015) offers another means by which lncRNAs can impact gene regulation and chromosome biology. If tandem arrays of repeats (simple, satellite, TE, etc.), such as those found in centromeres, pericentromeres, telomeres, and heterochromatin blocks, produce triplex structures, scaffolding and chromatin factor recruitment could impact regional chromosome function and/or sub-cellular localization. For example, rDNA promoter methylation and regional silencing of rDNA transcription is initiated by the recruitment of DNMT3B, and subsequent interactions with the nucleolar remodeling complex NorC, following triplex formation with an antisense RNA (Bierhoff et al. 2010; Schmitz et al. 2010).

Computational methods have led to the prediction of the possible sites in the human genome that could form RNA:DNA triplex structures (Buske et al. 2012; Goni et al. 2004; Jalali et al. 2017; Wu et al. 2007) indicating that at least one putative triplex site exists for each gene, promoter, and intergenic region. To avoid the isolation of RNA-DNA interactions formed through a protein intermediary, in vivo approaches to isolate RNA:DNA triplex structures should not rely on cross-linked samples. Rather, a recently described pair of methods (Senturk Cetin et al. 2019) removes free RNA from RNA that is bound to DNA through Hoogsteen pairing using a urea/NP40 extraction to isolate chromatin that is then treated with proteinase K to remove RNA bound to DNA via a protein intermediary. DNA:RNA triplex structures are further enriched using two complementary methods, paramagnetic bead selection and RNA immunoprecipitation via an anti-DNA antibody. Isolated RNA is then subjected to strand-specific RNA-seq and the sequencing data mapped back to the genome.

These genome-scale methods revealed that a surprising number of protein coding genes produced RNAs that associated with DNA in triplex structures (Senturk Cetin et al. 2019). These RNAs may represent noncoding isoforms of protein coding transcripts or other ncRNAs embedded within the transcript, such as miRNAs or antisense RNAs (Ayupe et al. 2015), or could be intragenic enhancer RNAs (Andersson et al. 2014; Cinghu et al. 2017). For these protein coding genes, the triplex may be fundamental to the gene’s function or transcriptional output (Senturk Cetin et al. 2019). In addition to these intragenic ncRNAs, an abundance of TEs and repeated elements were identified in these screens as triplex bound RNAs (Senturk Cetin et al. 2019), revealing the possibility that repeat-derived RNAs could interact with multiple genomic locations sharing sequence identity.

Given the observation that repeats within specific TEs can act as super-enhancers (Goni et al. 2004; Soibam 2017) or control nuclear localization of RNAs, such as SIRLOIN elements (Lubelsky and Ulitsky 2018), triplexes formed with repeated sequences could provide a potent means for repeat-bearing TEs to interact with DNA *in trans*. In support of this idea is the recent discovery that a defined, short motif is shared between *Xist* RNA and LINE1s in mouse and human that is predicted to mediate redundant lncRNA-triplex structures between *Xist* RNAs and X-linked LINEs during X-inactivation (Matsuno et al. 2019). Intriguingly, while a redundant UC/TC (r-UC/TC) motif was found in the two eutherian species, a redundant AG (r-AG) motifs was found to be shared between the putative marsupial X-inactivation mediating lncRNA, *RNA-on-the-silent X (Rsx)*, and LINEs within opossum. The lineage-specific convergence in redundant motif sequences shared between lncRNAs involved in X chromosome inactivation and X-linked LINEs may indicate that lncRNA-LINE triplexes are essential for inactivation of the X in females (Matsuno et al. 2019).

### Beyond RNA:DNA interactions

The identity of a specific RNA’s interacting partners can be revealed by screening the entire genome for those partners (also referred to as a ONE vs MANY approach). Three techniques employing the ONE vs MANY approach, ChIRP (chromatin isolation by RNA purification) (Chu et al. 2011), RAP (RNA antisense purification) (Engreitz et al. 2013), and CHART (capture hybridization analysis of RNA targets) (Simon et al. 2011), isolate all interacting partners for a specific RNA using biotinylated, complementary oligonucleotides for the RNA in cells that have been treated with cross linking reagents to allow isolation of nucleic acid-protein interactions. Where these applications vary are in the cross-linking reagent and chromatin treatments and in the design of the oligonucleotide probes for the target RNA (Simon 2016). Long probes are used in RAP and probes spanning the entire RNA (i.e., tiling) are used in both RAP and ChIRP, alleviating the need to predict accessible parts of an RNA molecule when in its folded form. CHART, on the other hand, utilizes RNAse H mapping to identify accessible regions of the RNA target for oligonucleotide probe design. Complexes isolated from these techniques can be further purified to identify RNA-protein partners via mass spectrophotometry (e.g., West et al. 2014), or the genomic locations of RNA interactions using deep sequencing (Chu et al. 2011; Engreitz et al. 2013; Simon et al. 2011). While useful in guiding the study ncRNAs of unknown function, these hybridization-based approaches also come with some caveats as artifacts such as hybridization to off target DNA or RNAs, directly or indirectly, can undermine precision of the data analysis (Simon 2016).

Alternative approaches for revealing RNA-chromatin interactions have been developed that do not rely on a known RNA and thus scan for all RNAs that may interact with chromatin. CheRNA-seq (chromatin-enriched RNA-seq), an approach to isolate chromatin-proximal RNAs, uses nuclear fractionation followed by RNA deep sequencing (Werner and Ruthenburg 2015) to separate soluble mRNAs and ncRNAs from RNAs that may function at the chromatin interface. Using a urea and Nonidet-P40 solution to separate released mRNAs from ternary complexes of RNA polymerase II and its DNA template (Bhatt et al. 2012; Wuarin and Schibler 1994), cheRNAs are isolated and sequenced at relatively high depth to ensure capture of low-abundance RNAs (Werner and Ruthenburg 2015).

Based on several of the same principles as the ONE vs MANY approaches, these MANY vs MANY approaches also begin with cross-linking RNA-protein complexes. Relying on proximity ligation, these methods employ a bivalent and biotinylated linker molecule that consists of single-stranded RNA at one end and double-stranded DNA at the other. Proximity ligation, wherein protein complexes that bring RNA and DNA together (i.e., on chromatin), is enabled by a bivalent linker containing a biotinylated bridge sequence, ligating the RNA portion to nascent RNA and the double-stranded DNA portion to proximal DNA. The MANY vs MANY techniques that rely on this type of proximity ligation, RNA-DNA heteroduplex capture include (Fig. 1b): MARGI (mapping RNA-genome interactions) (Sridhar et al. 2017), GRID-seq (global RNA interactions with DNA by deep sequencing) (Li et al. 2017), and ChAR-seq (chromatin associate RNA sequencing) (Bell et al. 2018). One technical component that distinguishes MARGI from ChAR and GRID is that the proximity ligation in the former is performed on extracted chromatin complexes (Sridhar et al. 2017), while in the latter two, proximity ligation is performed in situ on intact nuclei (Fig. 1b) (Bell et al. 2018; Li et al. 2017). Further distinguishing GRID and ChAR approaches is the post-ligation processing. GRID-seq includes a restriction enzyme digestion following reverse transcriptase conversion of the RNA-DNA duplex to a cDNA-DNA duplex. The targeted digestion 19–23 bp from the bridge sequence (this is done using the enzyme *MmeI* whose recognition sequence is within the bridge but cuts 18–20 bp away) allows size selection prior to sequencing to enrich for fragments containing RNA-DNA ligations (Fig. 1b, left) (Li et al. 2017). ChAR-seq, on the other hand, isolates 100-125 bp each of the DNA and cDNA sequences (Bell et al. 2018). The fivefold greater length of sequence obtained in ChAR-seq supports more accurate mapping to the reference, which can influence the interpretation of global RNA-seq data (Fig. 1b, right) (Li et al. 2017), particularly when repeats are considered.

From these collective approaches, a model of how transcription, transcripts, and chromatin remodeling are coordinated is emerging that indicates there is no single rule that defines lncRNA-chromatin interactions. For example, these studies confirmed the previous work demonstrating some lncRNAs interact *in cis* near their site of transcription while others work across larger regions or even across different chromosomes. Surprisingly, promoters/TSSs were found to have an association with *trans*-interacting RNAs (Li et al. 2017; Sridhar et al. 2017) while enhancers were found to associate with transcripts of their regulating gene (Li et al. 2017). Regions with *trans*-interacting RNA attachment were also correlated with open chromatin histone marks, H3K27ac and H3K4me3 (Sridhar et al. 2017), but this correlation was not consistent across all RNAs. snoRNAs interactions, for example, are enriched for marks of heterochromatin rather than active transcription (Bell et al. 2018).

The application of ChAR-seq to *Drosophila* cells indicated that transcription-associated RNAs are enriched at TAD boundaries, linking RNA-chromatin interactions to 3D genome architecture (Bell et al. 2018). In fact, a recently described technique RADICL-seq (RNA and DNA interacting complexes ligated and sequenced) was applied to mouse cells, revealing an enrichment of RNA-chromatin interactions at TAD boundaries specifically associated with TEs (Bonetti et al. 2019), indicating such interactions may be a conserved mechanism for the control of genome organization.

## The final frontier: incorporation of repeats and TEs in ncRNA data analyses

The descriptors for genome-scale studies often include “all” rather than “many,” as used in this review. However, the use of “all” is misleading as it implies that the entirety of the genome serves as a reference for mapping NGS datasets. Rather, it is understood that these data analyses are contemporaneous with *available* genome sequence. Herein lies one of the major challenges for the field: *how do we obtain a comprehensive understanding of RNA-chromatin relationships, particularly when ncRNAs containing, or derived from, repeats are considered, when we have yet to fully annotate the complete sequence content of the genome?* Reference genomes for most model species are not chromosome-level to the extent that all scaffolds are provided with both chromosomal assignment and linear arrangement (Lewin et al. 2019). An estimated 10% of the human reference genome (hg38), considered to be one of the best eukaryotic genome assemblies to date, remains on orphan scaffolds enriched for repeat-dense regions of the genome, such as rDNA loci, centromeres, interstitial repeat clusters, telomeres, and pericentric regions (Altemose et al. 2014; Miga 2015; Rosenbloom et al. 2015).

**Fig. 2:**
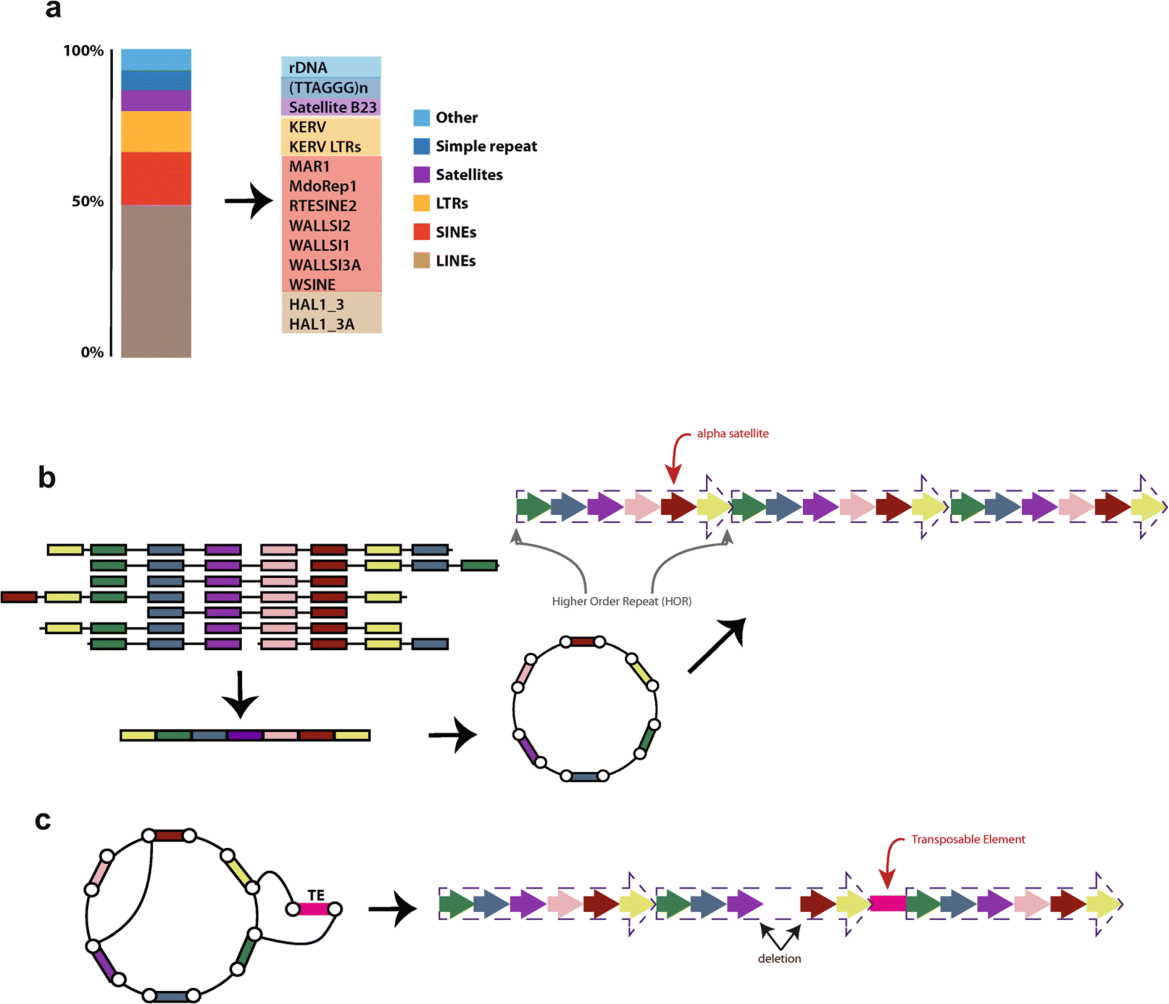
Examples of methods used to study repeats in the absence of a genome assembly. **a** Repeat Masker applied to raw sequencing data provides details on overall frequency of repeats by class (left) and specific type (right). **c** The linear order of highly repeated sequences, such as human alpha satellites found in centromeres, can be inferred from whole genome shotgun data (paired end sequencing). The resulting graphical model illustrates the frequency and order of satellite sequences (colored blocks). From the circular model, a linear arrangement of centromere satellites (colored arrows) can be inferred, including higher order repeat arrays (dotted arrows). **c** Variations within centromere arrays, such as deletions and insertions, can be captured with the graphical model approach

The short-read lengths inherent to modern high-depth sequencing technologies, coupled with the difficulty in assigning highly similar repeats to a specific location in a reference genome, are major limitations to closing gaps in genome assemblies for most complex eukaryotic genomes. Techniques such as Hi-C (Lieberman-Aiden et al. 2009) greatly improve the ability to assign contigs to chromosomes (Burton et al. 2013; Kaplan and Dekker 2013; Marie-Nelly et al. 2014), but are not capable of building full, chromosome-scale scaffolds on their own (Lewin et al. 2019). Despite these seemingly insurmountable challenges, researchers have developed an ever-growing set of tools to both catalog and analyze repeats across the genome. For example, RepeatMasker is used to classify repeats based on a compiled database (such as Repbase (Jurka 2000)) using gapped aligners, affording the ability to classify highly variable sequences (Smit et al. 2015). While traditionally considered for repeat annotations in genome assemblies, this tool can be applied to HTS reads, regardless of their source (RNA or DNA from various applications, as described in this review) (Fig. 2a).

In like fashion, if a particular repeat class is known, any sequences within HTS datasets with identity to this class can be isolated from a pool of sequences and a k-mer approach can be used to define the phylogenetic relationships among repeats (Smalec et al. 2019) or derive graphical models of repeat content (Miga et al. 2014; Rosenbloom et al. 2015). For example, the linear order and frequency of individual repeats within large tandem arrays, exemplified by alpha satellites in human centromeres, was inferred from linked pairs of sequencing reads from whole genome shotgun data (Fig. 2b) (Miga et al. 2014; Rosenbloom et al. 2015). In addition, the frequency and classification of transposable element insertions into repeat arrays can be assessed using this graphical model approach (Fig. 2c).

**Fig. 3:**
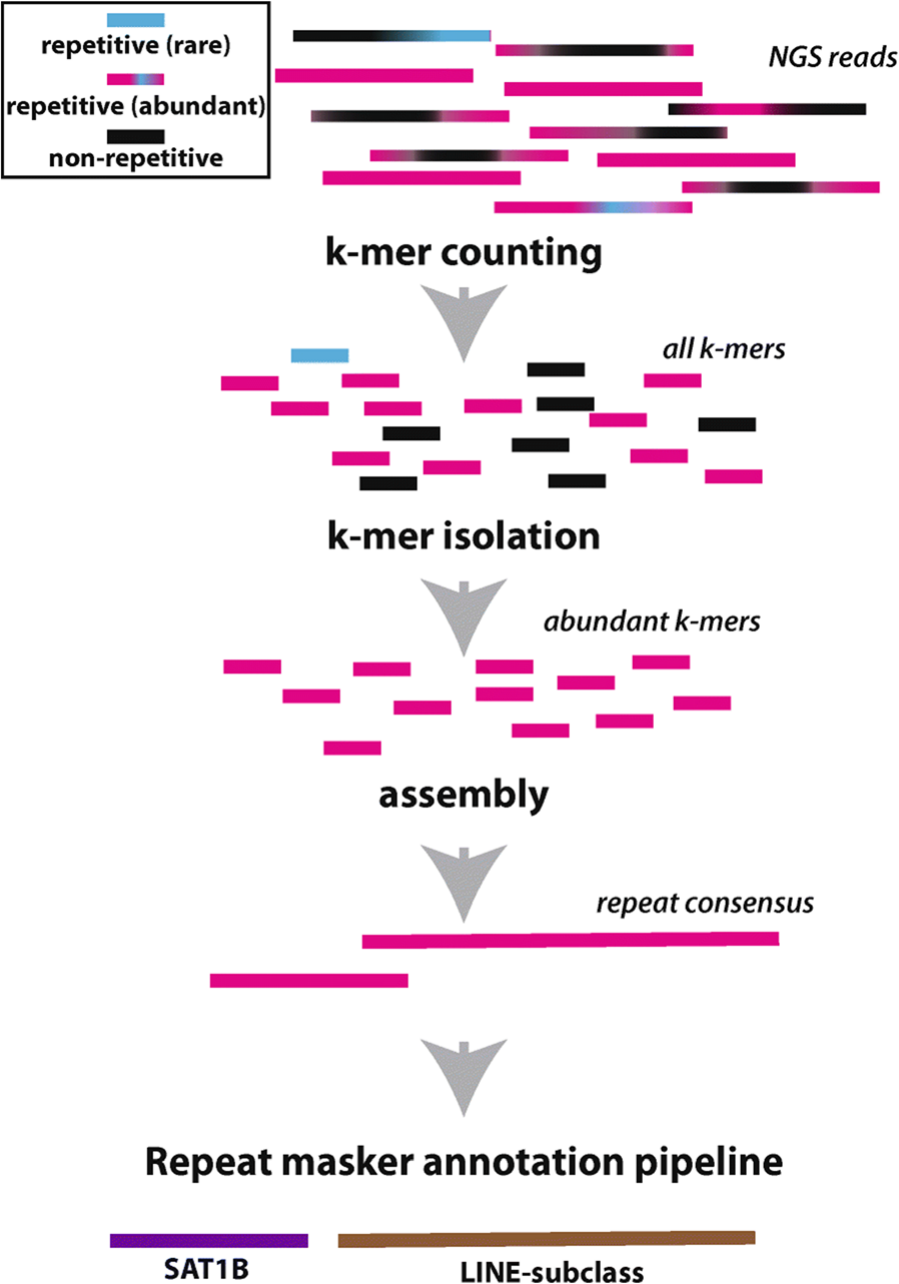
RepARK uses k-mers to build a de novo assembly of repeats that can be further annotated for specific repeat type

In the absence of a complete, telomere-to-telomere genome assembly, other approaches can be applied to study the contribution of repeats to the RNA-chromatin relationship. Current mapping tools, such as BWA and Bowtie2, (Langmead and Salzberg 2012; Li 2013), are typically implemented to report unique mappers only; in other words, sequencing reads that map to more than 1 location in the queried genome are ignored. In doing so, the contribution of repeats are often overlooked or minimized. To complement standard mapping strategies, HTS datasets can be explored for repeat content via genome independent methods. For example, sequencing reads can be annotated for repeat content using repeat masking pipelines to reveal the types of repeats and their frequency within a given HTS dataset. K-mer based approaches can also be used to classify reads into specific repeat groups (Lefebvre et al. 2003; Marcais and Kingsford 2011). Approaches that derive de novo assemblies from HTS data have also been developed, such as RepARK (Koch et al. 2014) (Fig. 3), REPdenovo (Chu et al. 2016), and ChIPtigs from ChIP-seq data (He et al. 2015). These methods rely on k-mers rather than alignments to build contigs, but in doing so less-frequent and rare k-mers may be lost in the final assembled contigs. While none of these methods offer a full replacement for a reference genome, they illuminate regions that are either missing, or highly variable, when compared to a single reference genome, such as those enriched in TEs, satellites and/or tandem arrays.

The arrival of long-read sequencing technologies in the genome sequencing market has provided a boost to the initiatives to derive genome assemblies that include repeats, particularly those with relevance to chromosome segregation. For example, the genome sequence of the koala, based on ~ 58× PacBio long-read sequencing and polishing with 30× Illumina short-read sequencing, afforded assembly of scaffolds that contained centromeres (Johnson et al. 2018). These scaffolds were functionally annotated with ChIP-seq data for a pool of centromere-binding proteins, revealing that transposable elements are a major contributor to centromere identity in this species (Johnson et al. 2018). In Drosophila, centromere scaffolds were assembled with the aid of long-read data from PacBio and chromosome assignment was achieved using oligo-paints derived from these assemblies (Chang et al. 2019). The annotations of repeats containing centromeric histones using a combination of ChIP-seq and ChIP-tig analyses showed that islands of transposable elements within satellite arrays define chromosome-specific centromere identity in Drosophila (Chang et al. 2019).

### Where do we go from here?

The combination of long-read sequencing data (i.e., Oxford Nanopore, PacBio) and applications such as Hi-C, accompanied by increasingly accessible high-coverage short-read sequencing are supporting efforts to complete telomere-to-telomere (T2T) assemblies for a reference human genome. Successes in this approach have been realized for the X chromosome (Miga et al. 2019), and are being expanded to the entire human genome (Miga et al. 2019). New computational tools (e.g., Bongartz 2019; Russo et al. 2019; Shafin et al. 2019) and assembly improvements for model species are facilitating additional analyses with existing “-seq” datasets from diverse applications. Moreover, genome-scale applications developed for short-read NGS technologies are being modified to incorporate long-read sequencing to enable more accurate mapping with the inclusion of junctions between repeats and unique sequences and the assembly of tandem arrays of repeats.

Such advances will enable a full appreciation of the dynamic and diverse RNA-chromatin relationships that exist in eukaryotic genomes. However, a major challenge will be to “carryover” exiting datasets developed to study RNA-chromatin interactions to new assemblies and repeat annotation pipelines as they emerge. Furthermore, the diversity of genomes across individuals within a population should be incorporated into studies exploring the role of ncRNAs in instability and disease. The lack of T2T-scale genomes that support comprehensive comparative approaches must be overcome (Doolittle 2018; Lewin et al. 2018) to fully appreciate conserved RNA-chromatin functions as well as divergent functions that enable evolutionary novelty (Kapusta et al. 2013). This is an exciting time where we are witnessing a re-emergence of the synergy of RNA biology and chromosome biology through innovations in genomics.

## Abbreviations

ATR: Ataxia telangiectasia mutated and Rad3-related
bis-DRIP: Bisulfide DNA:RNA immunoprecipitation
caRNAs: Chromatin-associated RNAs
ChAR-seq: Chromatin associate RNA sequencing
CHART: Capture hybridization analysis of RNA targets
cheRNA-seq: Chromatin-enriched RNA-seq
ChIRP: Chromatin isolation by RNA purification
ChRO-seq: Chromatin run-on sequencing
CUT&RUN: Cleavage under targets and release using nuclease
DRIP-seq: DNA:RNA immunoprecipitation
DRIVE: DNA:RNA in vitro enrichment
GRID-seq: Global RNA interactions with DNA by deep sequencing
GRO-seq: Global run-on sequencing
HTS: High-throughput sequencing
lincRNA: Long intergenic noncoding RNAs
lncRNA: Long noncoding RNA
MARGI: Mapping RNA genome interactions
miRNAs: MicroRNAs
ncRNA: Noncoding RNA
piRNAs: Piwi-interacting RNAs
PRO-cap: Precision run-on sequencing of capped RNA
PRO-seq: Precision run-on sequencing
RADICL-seq: RNA- and DNA-interacting complexes ligated and sequenced
RAP: RNA antisense purification
R-ChIP: R-loop chromatin enrichment
RDIP: RNA:DNA immunoprecipitation
RIDLs: Repeat insertion domains of lncRNAs
R-loop: RNA moiety loop
RPA: Replication protein A
S1DRIP: S1-nuclease DRIP-seq
SIRLOIN: SINE-derived nuclear organization
snoRNA: Small nucleolar RNAs
TEs: Transposable elements
tRNAs: Transfer RNAs
TSS: Transcription start site
