# Correction to: Seq’ing identity and function in a repeat-derived noncoding RNA world

**DOI:** 10.1007/s10577-021-09665-2

**Published:** 2021-06-24

**Authors:** Rachel J. O’Neill

**Affiliations:** 1 Institute for Systems Genomics, University of Connecticut, Storrs, CT 06269 USA; 2 Department of Molecular and Cell Biology, University of Connecticut, Storrs, CT 06269 USA; 3 Department of Genetics and Genome Sciences, University of Connecticut Health Center, Farmington, CT 06030 USA

**Correction to: Chromosome Res (2020) 28:111–127**

10.1007/s10577-020-09628-z

